# Effects of Thymoquinone on radiation enteritis in mice

**DOI:** 10.1038/s41598-018-33214-3

**Published:** 2018-10-11

**Authors:** Qinlian Hou, Linlin Liu, Yinping Dong, Jing Wu, Liqing Du, Hui Dong, Deguan Li

**Affiliations:** 1 Institute of Radiation Medicine, Chinese Academy of Medical Science and Peking Union Medical College, Tianjin Key Laboratory of Radiation Medicine and Molecular Nuclear Medicine, Tianjin, 300192 China; 2 Tianjin Hospital of Itcwm Nankai Hospital, Department of prediatrics, Tianjin, 300100 China

## Abstract

Radiation enteritis is an old but emerging question induced by the application of radiation. However, no effective drugs for radiation enteritis in clinic. In this study, we found that thymoquinone (TQ) could mitigate intestinal damages induced by irradiation. After exposure to irradiation, TQ-treated improved the irradiated mice survival rate, ameliorated intestinal injury and increased the numbers of intestinal crypts. Furthermore, Lgr5^+^ ISCs and their daughter cells, including Vil1^+^ enterocytes, Ki67^+^ cells and lysozyme^+^ Paneth cells, were all significantly increased with TQ treatment. In addition, P53, γH2AX, caspase8, caspase9 and caspase3 expression were all reduced by TQ. Our data showed that TQ modulated DNA damages and decreased the apoptosis in the small intestine. TQ might be used for radiation enteritis treatment.

## Introduction

Evidence has shown the hematopoietic system and small intestine are the major injury sites during radiotherapy and radiation accident for their higher radiation sensitivity^1^. Acute radiation enteritis is the result of the intestinal epithelium, especially crypt stem cells exposed to radiation^2^. But there are still no widely approved drugs or methods for reducing the occurrence or severity of acute radiation enteritis. Therefore, strategies to prevent intestinal irradiation injury are required both to improve the effectiveness and outcome after therapeutic radiation.

The small intestine epithelium epithelial cells would be degeneration, necrosis and apoptosis, etc., villous epithelial renewal blocked, intestinal barrier function lost, even individual deaths induced by bacteremia and toxemia when receiving radiation^3–5^. Apoptosis^6^ mediated by the p53 overexpression^7,8^ and the Bcl-2 decrease^9^ plays an important role in the acute radiation enteritis. The following mucositis interferes with the intestinal barrier dysfunction may lead to the translocation of luminal bacteria^10,11^.

Thymoquinone(TQ, 2-Isopropyl-5-methyl-1,4-benzo-quinone, C_10_H_12_O_2_), extracted from black cumin (Nigella sativa, NS) seed oil^12^, which exerts powerful anti-inflammatory, antioxidant and antitumor activities. TQ has been used to treat various diseases, while been minimally toxic to normal cells^13^. TQ shows protective effects on several animal models of inflammatory response. TQ also can prevent and ameliorate Dextran Sulfate Sodium (DSS) induced Colitis in mice^14^. But the effects of TQ on the radiation enteritis have not been reported.

In this study, we firstly report that TQ has a protective effect on radiation enteritis, and TQ can decrease intestinal cells apoptosis by inhibiting P53 pathway.

## Results

### TQ improves mice survival rate

**Figure 1:**
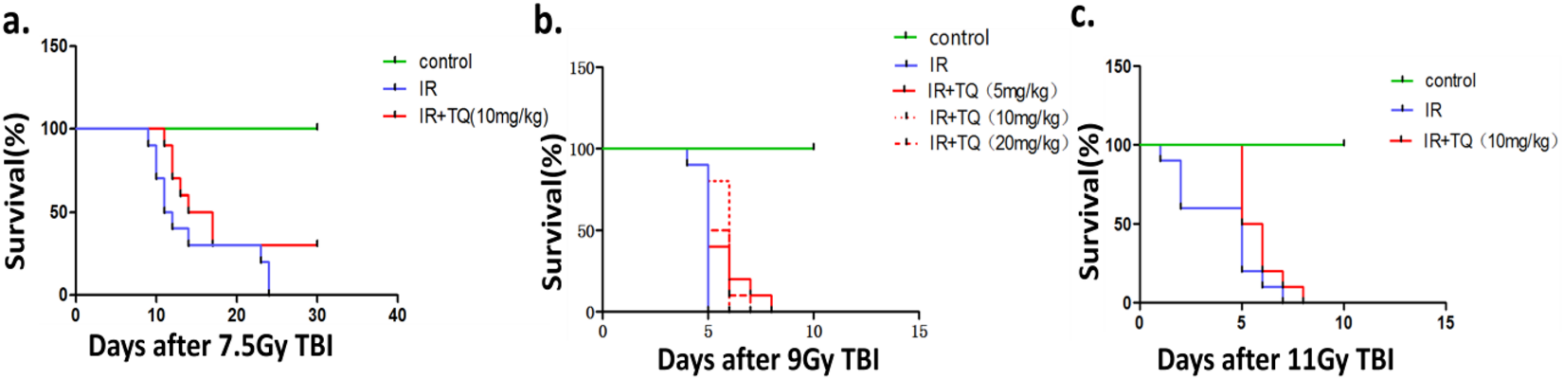
TQ protects mice from radiation-induced mortality. (**a**) Survival curve of C57BL/6 mice treated with saline or TQ after 7.5 Gy TBI. The number of animals in each treatment group is shown in parentheses. (**b**) Survival curve of C57BL/6 mice treated with saline or different concentration TQ after 9.0 Gy TBI. (**c**) Survival curve of C57BL/6 mice treated with saline or TQ after 11.0 Gy TBI.

To assess the protective effect of TQ on Total body irradiation (TBI)-induced lethality in mice, the survival rates of mice exposed to different doses TBI were observed. After 7.5 Gy TBI (Fig. 1a), all mice died at at 24 days, compared with TQ treated group having 30% survival beyond 30 days. We treated the mice with three doses of TQ (5 mg/kg, 10 mg/kg and 20 mg/kg), and then the mice were exposed to 9.0 Gy TBI (Fig. 1b). All doses could improve the mice survival rate compared to the saline treated group. There was 80% mortality in saline treated mice at 6 days after 11.0 Gy TBI (Fig. 1c), while 50% mice survival in TQ treated mice, suggesting that TQ may have a protective effect on mice after radiation. These results indicate that TQ effectively mitigates the TBI-induced lethality in mice.

### TQ attenuates structural damages of the small intestine and changes the levels of GSH, SOD and MDA

**Figure 2:**
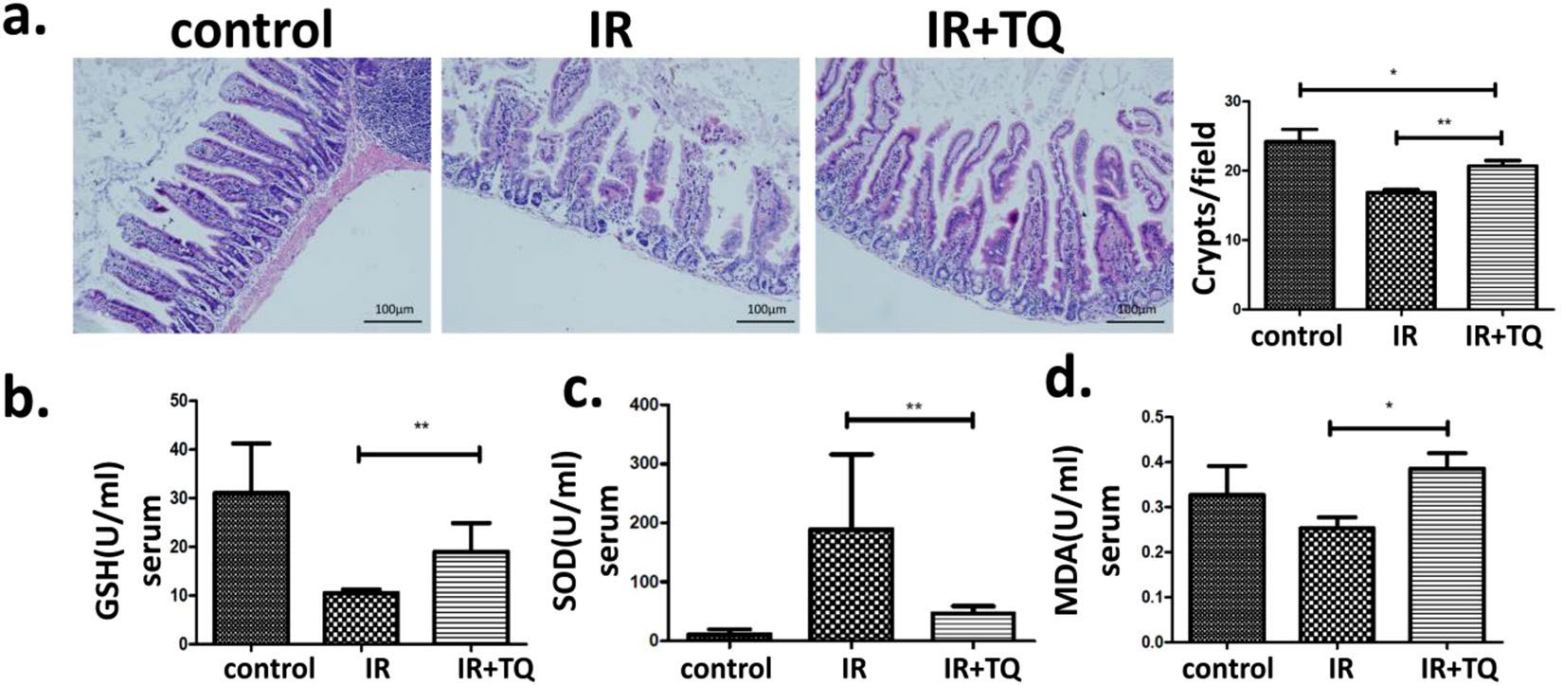
TQ protects mice small intestinal, and changes the levels of GSH, SOD and MDA. (**a**) Saline-treated mice and TQ-treated mice after TBI. In H&E-stained sections, crypt-villus architecture of TQ-treated mice was well preserved, and the number of crypts had significantly increased by days 3.5 post-radiation compared with saline-treated mice. The numbers of crypts per length were analyzed. (**b**) GSH of the different groups. (**c**) SOD of the different groups. (**d**) MDA of the different groups. Results conclude from at least fifteen regions and shown as the mean ± SEM. **p < 0.01. bars, 100 μm.

To examine the putative role of TQ against radiation intestinal injury, the histological manifestations were compared. At 3.5 day post 9.0 Gy TBI, mucosal destructions such as villous denudation and crypt atrophy, were observed. The crypt-villi structures of the small intestine were preserved in TQ treatment group (Fig. 2a).

Major antioxidant defenses include antioxidant scavengers, such as total glutathione (GSH) and Superoxide Dismutase (SOD). GSH and SOD can remove lipid peroxides, reducing lipid peroxidation by regulating the balance of body’s oxidation and antioxidant. An increase in lipid peroxidation products (MDA), which is a lipid peroxidation end-product, indicates reduced antioxidant capacity^15,16^. Total SOD, GSH and MDA activities were measured in liver tissue and serum.

Compared with IR, the GSH levels in liver tissue of the TQ group increased (Fig. 2b), the SOD levels in serum increased (Fig. 2c), the MDA levels in serum of the TQ group reduced (Fig. 2d), suggesting that TQ treatment significantly increased ROS scavenge activity.

### TQ enhances Lgr5^+^ ISC survival, promotes Paneth cell survival, increases the Ki67^+^ cells and vill^+^ enterocytes proliferation

**Figure 3:**
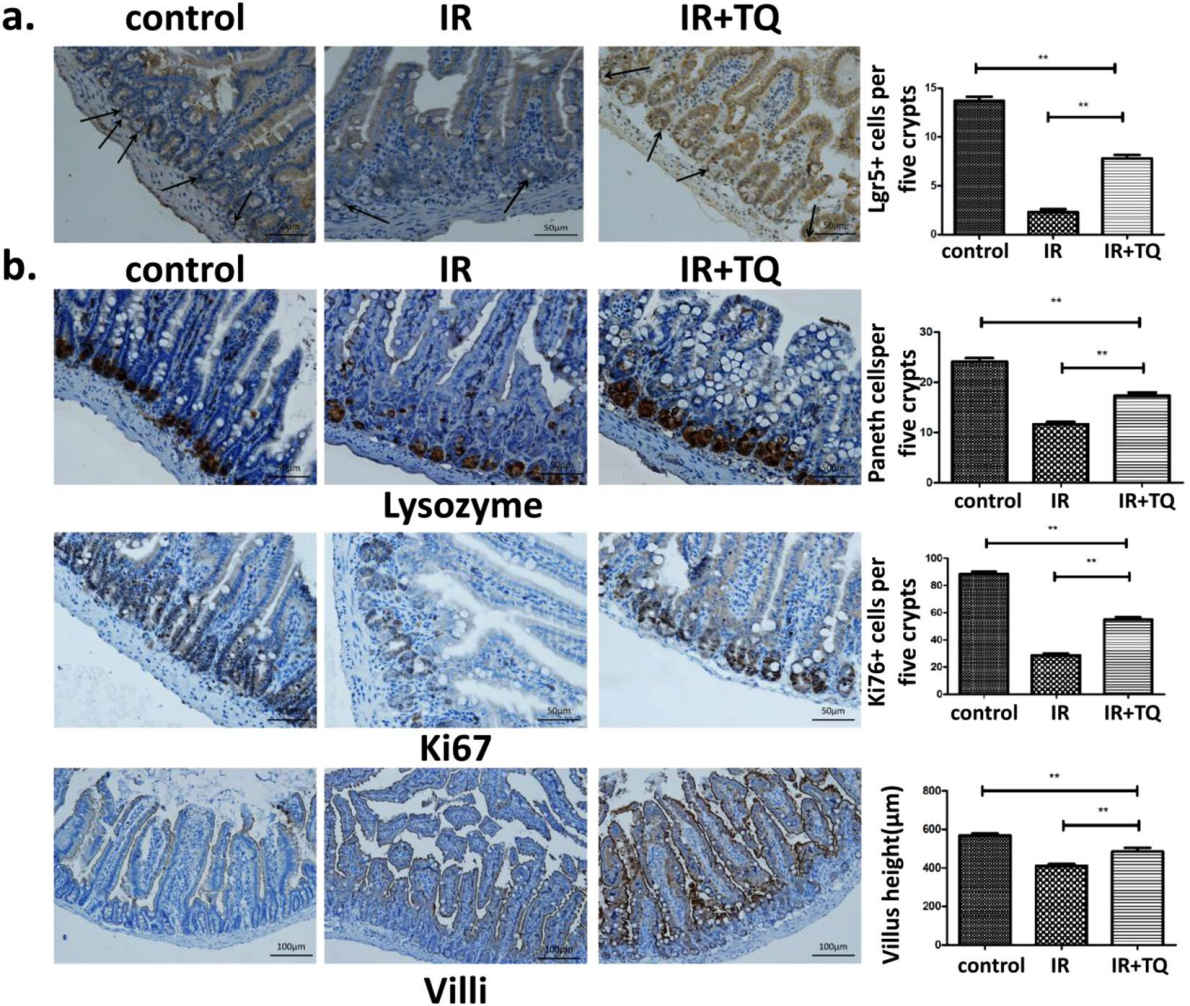
TQ enhances Lgr5^+^ ISCs, Paneth cell, Ki67^+^ cells, vill^+^ enterocytes. (**a**) After radiation 3.5 days. Lgr5 + cells were counted in five crypts. (**b**) TQ promotes Paneth cell survival and increases the proliferation of Ki67^+^ cells and vill^+^ enterocytes. After TBI 3.5 day, small intestinal tissues were collected and immunostained to observe Ki67^+^ cells, lysozymes, and vill^+^ enterocytes. Results conclude from at least fifteen regions and shown as the mean ± SEM. **p < 0.01 bars, 50 μm and 100 μm.

Lgr5^+^ ISCs have been shown to be indispensable for intestinal regeneration following radiation injury^17,18^. After radiation 3.5 day, the Lgr5^+^ ISCs numbers in IR mice were significantly decreased compared with the control, while the numbers of Lgr5^+^ ISCs were significantly increased in mice treated with TQ compared to the IR group (Fig. 3a). In addition, the Lgr5 protein levels in the small intestine were elevated, which was consistent with Lgr5 staining results (Supplementary Fig. 1).

Paneth cells located at the bottom of intestinal crypts produce lysozymes^19^. The changes of Paneth cells were investigated at 3.5 days after 9.0 Gy TBI (Fig. 3b). Compared to the control group, the numbers of paneth cells in IR groups significantly decreased the TQ elevated the numbers of intestinal Paneth cells compared to the IR group.

Ki67^+^ immunostaining identifies transient amplifying cells, which is seemed as the regenerative response of the epithelial layer. After 9.0 Gy TBI 3.5 day, there was a significant reduction in Ki67^+^ cells. TQ treatment resulted in an increase in Ki67 staining (Fig. 3b). These data indicated that TQ could promote cell proliferation.

Radiation caused villi damage, and reduced vill^+^ enterocytes. Compared to IR group, TQ significantly increased the vill^+^ cells (Fig. 3b). TQ could improve the protection of vill^+^ enterocytes in radiation-induced injury.

### TQ decreases P53 and γH2AX expression

**Figure 4:**
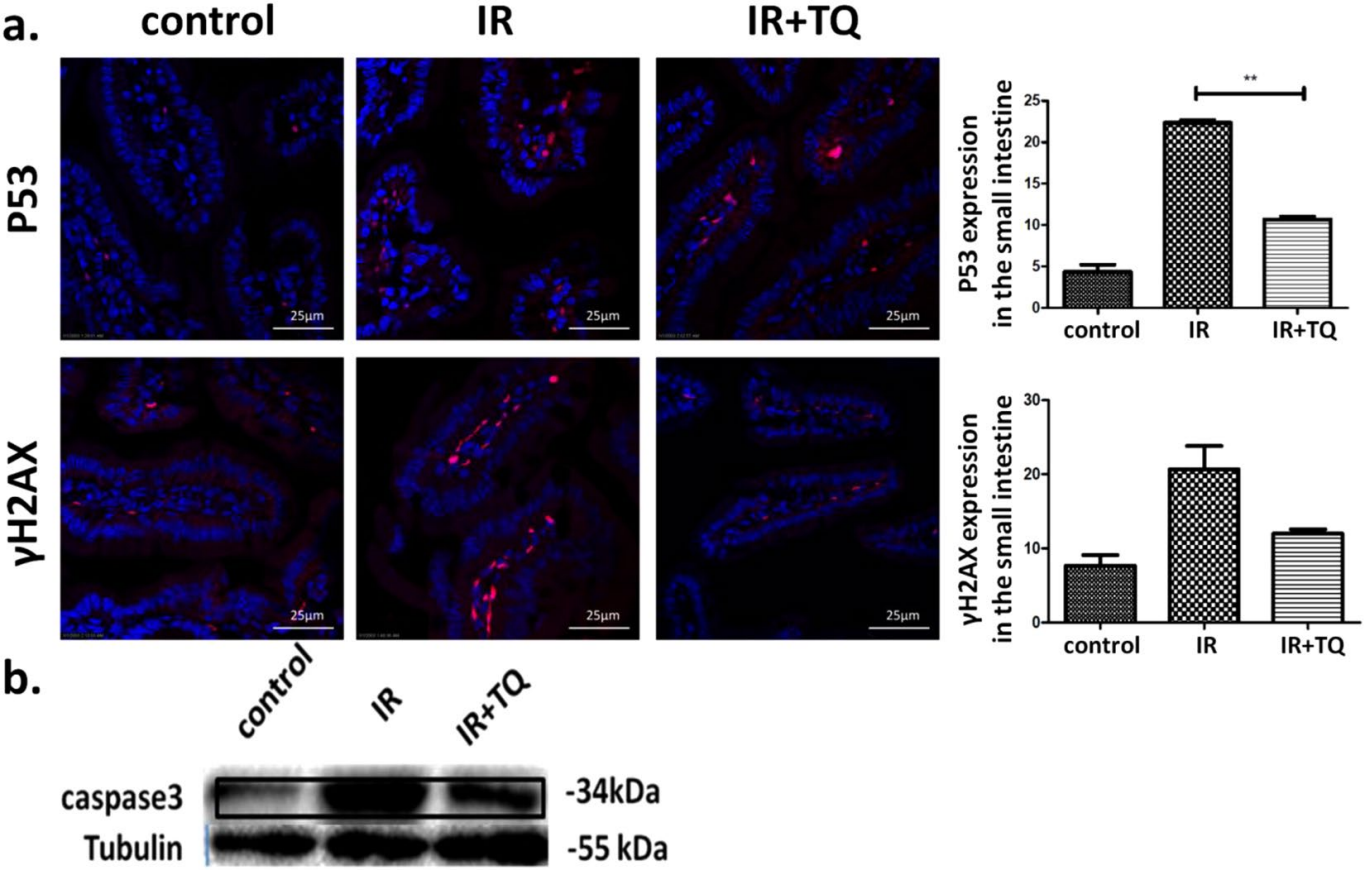
TQ reduces P53 and γH2AX expression. (**a**) Immunofluorescent staining of p53, γH2AX, and DAPI in the small intestine with different treatment, compared with control. (**b**) The protein level of caspase3 in small intestine was measured by Western blot. Results conclude from at least fifteen regions and shown as the mean ± SEM. **p < 0.01 bars, 10 μm.

To determine whether p53 pathway was regulated by TQ, p53 were detected. IR mice expressed P53 in the small intestine had a fourfold increase compared to those from control mice. However, TQ-treated mice expressed P53 in the small intestine half reduced compared to those from saline-treated mice (Fig. 4a).

Meanwhile, γH2AX, which usually couples with DNA damage responses (DDR), has been widely regarded as a marker of DNA damage and utilized in pre-clinical drug development and clinical studies^20,21^. The γH2AX expression of IR group in the small intestine had twice increase compared to those from control mice. TQ-treated mice expressed γH2AX in the small intestine had less than half reduction compared to those from IR group (Fig. 4a). In addition, the expression of Bax in the intestinal crypts cells was also evaluated, which was consistent with others (Supplementary Fig. 1).

### TQ decreases the apoptosis of the small intestine

**Figure 5:**
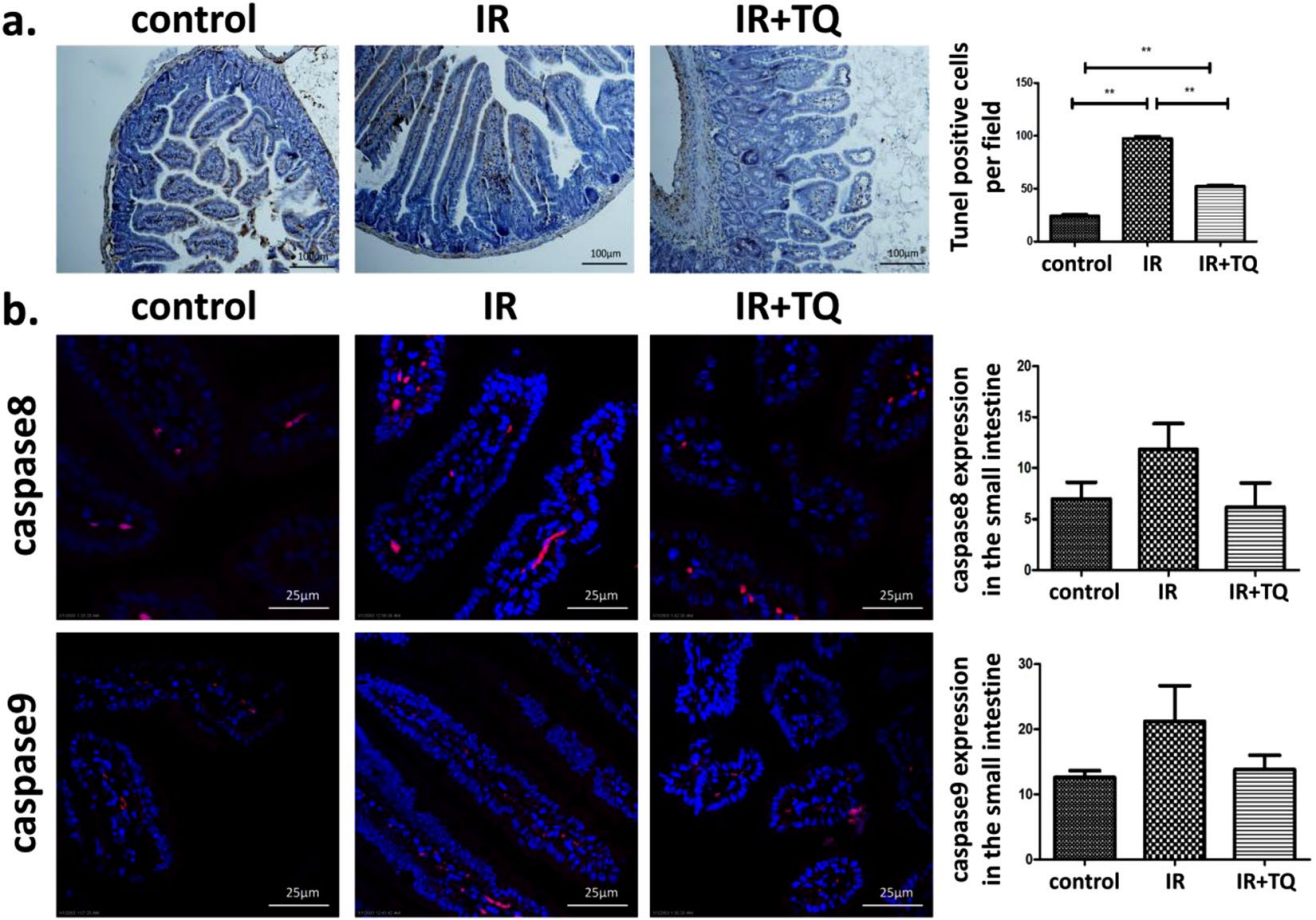
TQ reduces apoptosis after radiation. (**a**) TQ reduced the radiation-induced apoptosis. Apoptosis was analyzed by TUNEL assays. The number of TUNEL^+^ cells was counted. (**b**) Immunofluorescent staining of caspase8, caspase9, and DAPI in the small intestine with different treatment, compared with control. Results conclude from at least fifteen regions and shown as the mean ± SEM. **p < 0.01. bars, 100 μm and 10 μm.

To further validate our observations, we evaluated the apoptosis in the small intestine by TUNEL assay (Fig. 5a). The results showed that TQ could prevent radiation-induced intestinal damage by apoptosis inhibition.

After 3.5 days radiation there was a significant increase in caspase8 (Fig. 5b) and caspase9 (Fig. 5c) expression in the IR mice compared to the control. TQ treatment resulted in a reduction in caspase8 and caspase9 staining. In addition, the caspase3 expression in the small intestine was also determined (Fig. 4b). These data showed that TQ could inhibit caspase cascade activated by radiation.

## Discussion

The efficiency of agents to prevent or mitigate radiation-induced injuries is crucial for improving radiotherapy effects and response to radiation accident^22,23^. In this study, TQ treatment increased the irradiated mice survival time and mitigated the acute radiation enteritis. These effects may be owing to the inhibitions of cell apoptosis and DNA damages.

Under physiological, TQ could protect the visceral and structural of the small intestine in the mice. Ionizing radiation causes acute radiation syndrome of gastrointestinal tract by Lgr5^+^ ISCs reduction. The crypt base columnar cells reside at the base of the crypt, and Lgr5^+^ crypt base columnar cells are commonly seemed as the intestinal stem cells^18,19,24^. After TQ treatment, Lgr5^+^ ISCs augment indicated that TQ could promote the survival of Lgr5^+^ ISCs. The expression of lysozymes in Paneth cells decreased after radiation^25,26^. While the expression of lysozymes in TQ treated group was significantly elevated compared to that of mice in control group. In addition, both Ki67^+^ cells and vill^+^ enterocytes numbers were also elevated after TQ treatment, which suggested that TQ decreased the radiation injury on the Paneth cells and enterocytes^27–29^. The GSH level was measured to verify the role of TQ in oxidative stress, which disrupts DNA damage and normal cellular signaling and is involved in various clinical diseases, particularly cancer^30–32^. The GSH levels in liver tissue were significantly lower in the IR group than in the TQ group. SOD can remove lipid peroxides, play a vital role for reducing lipid peroxidation by regulating the balance of body’s oxidation and antioxidant. The SOD levels in serum were significantly lower in the IR group than in the TQ group. The amount of MDA who can damage biofilm often be reflected in the extent of lipid peroxidation. The MDA levels in serum were significantly higher in the IR group than in the TQ group. The data suggested that TQ might protect intestinal radiation damages by enhancing antioxidant ability.

Radiation produces DNA damage directly through reactive oxygen species^33^, and destroys the expression of proteins in cells^34^, activating p53^4,33,35,36^. In addition, DNA injury induced by IR was determined by γH2AX phosphorylation, and this was used as an indicator for quantifying DNA double-strand breaks^37^. Recent studies suggest that p53 in intestinal epithelial cells principally controls radiation-induced GI toxicity in mice, independently of apoptosis^4^. TQ reduces the expression of p53 and Bax compared with IR group. The data suggested that TQ may mitigate radiation induced DNA damage by p53 inhibition^38–41^.

Previous researches have shown p53 induced cell apoptosis by activating caspase cascade^42^. Caspase3 are effector caspases that can be activated by initiator caspases (caspase8, caspase9) to cause cell apoptosis^43,44^. In this study, TQ treatment could inhibit the expression of caspase8, and caspase9 at 3.5 days after 9.0 Gy TBI. Caspase3 expression also declined in the TQ-treated group. These results indicated that TQ could regulate caspase apoptotic pathways induced by radiation.

In conclusion, TQ treatment decreased the severity and duration of radiation-induced enteritis by reducing tissue injury, cell apoptosis and DNA damages. TQ might be used as the radiation protector.

## Materials and Methods

### Radiation

Irradiation was performed using a ^137^Cs source housed in an Exposure Instrument Gammacell-40 (Atomic Energy of Canada Lim, Chalk River, ON, Canada) at a dose-rate of 1.0 Gy per minute. Sham-irradiated mice were treated similarly to the irradiated mice but without exposure to IR. After irradiation, the mice were returned to the animal facility for daily observation and treatment as described below. The mice were exposed to 7.5 Gy, 9.0 Gy and 11.0 Gy TBI in the survival experiments, each group has 10 mice^45^, and 9.0 Gy TBI in the remaining experiments^46^.

The mice in survival experiments were randomly assigned to 3 treatment groups: control, saline + 7.5 Gy TBI, 10 mg/kg TQ + 7.5 Gy TBI. For the TQ treatment, the mice were administered 0.2 mL of solution by gavage 1 times over the 1 day prior to irradiation. The control and 7.5 Gy TBI groups were treated with saline similarly to the procedure described for the TQ treatments. All the mice in irradiated groups were irradiated after the treatment. The same goes for 9.0 Gy and 11.0 Gy.

The 15 mice in the remaining experiments were randomly assigned to 3 treatment groups: control, saline + 9.0 Gy TBI, 10 mg/kg TQ + 9.0 Gy TBI. The mice were treated as described above and were killed 3.5 days after exposure to irradiation.

All mice in this study were of a pure C57BL/6 genetic background and separated into groups randomly, treated according to the guidelines established by the National Institutes of Health Guide (NIH) for use. All experiments were done in accordance with procedures approved by the Daegu-Gyeongbuk Medical Innovation Foundation (DGMIF) Institutional Animal Care and Use Committee (IACUC). All procedures and animals handlings were performed following the ethical guidelines for animal studies.

### GSH, SOD and MDA index detection

GSH, SOD and MDA activity were all measured using the method according to the manufacturer’s protocols (Nanjing Jiancheng Corp., China).

### Histological Analysis

At three-day after IR, mice were sacrificed and the small intestines were collected and stained with hematoxylin-eosin (H&E) and analyzed under a microscope. For morphological analysis, six circular transverse sections were analyzed per mice in a blind manner from coded digital H&E stained photographs to measure the villi length and crypt number by using the ImageJ 1.37 software.

### Immunohistochemistry

Mice were killed at 3.5 days WBI, and the small intestine was fixed with neutral formalin. Then the tissues were dehydrated and embedded with paraffin. Then the sections were boiled in 10 mM/L citrate buffer solution (pH 9.0) for antigen retrieval according to the standard procedures. After antigen retrieval, the sections were incubated with serum for 1 h at room temperature to block non-specific antigen-binding sites, and then with anti-Lgr5 antibody (1:50 dilution, Abcam, Cambridge, MA, USA), anti-Ki67 antibody (1:300 dilution, Novus a biotechne brand), anti-lysozyme (1:800 dilution, Abcam, Cambridge, MA, USA) or anti-villi (1:800 dilution, Abcam, Cambridge, MA, USA) overnight at 4 °C. Sections were then incubated in secondary antibody for 30 min at 37 °C. Positive cells were detected using DAB kit (Sigma Aldrich). The images were captured and positive staining was quantified objectively by the IPP software as described previously in a blinded fashion.

### TUNEL assay

Sections (3 μm thick) were treated using the method according to the manufacturer’s protocols (Roche, Mannheim, Germany). At last, sections were analyzed by light microscope.

### Immunofluorescence

In brief, sections(3 μm thick) were dewaxed and treated with citrate buffer. After antigen retrieval, sections were treated, blocked and stained with p53, rH2AX, caspase8, and caspase9 antibodies for 1 hour at 37 °C. Sections were washed and incubated with an appropriate dilution of secondary antibodies conjugated with Alexa fluor 488 and 594 respectively, for 1 h at RT. Stained Sections were washed and viewed under a fluorescence microscope equipped with the Nikon Metamorph digital imaging system. Nuclei were visualized by using DAPI as a counter stain.

### Isolation of Intestinal Crypts Cells

The method of isolating intestinal crypts was described^47,48^. Briefly, the small intestines were chopped into small pieces and then placed into cold crypt chelating buffer for 30 min. After rinsing, the fragments were re-suspended and collected.

### Western blots

Protein was extracted from small intestines incubated in ice-cold lysis buffer (Solarbio Science and Technology, Beijing, China). Protein concentrations were quantified using a BCA protein kit (Beyotime, Shanghai, China). Samples containing equal amounts of protein (20 μg) were mixed with loading buffer containing 5% 2-mercaptoethanol, heated for 10 min at 95 °C, and loaded onto a 10% SDS-PAGE gel. After electrophoresing the proteins along the gel, the proteins were then transferred to polyvinylidene difluoride membranes. These membranes were blocked with 5% milk and 0.1% Tween 20 in Tris-buffered saline, and then incubated overnight at 4 °C with primary antibody, that is, anti- Lgr5 (1:1000 dilition, Abcam, Cambridge, MA, USA), anti-caspase3 (1:1000 dilition, ruiyingbio, Suzhou, China), anti-Bax (1:1000 dilition, Ruiyingbio, Suzhou, China) or anti-tubulin (1:2500 dilition, SANYING, Wuhan, China), followed by the appropriate horseradish peroxide-conjugated secondary antibody at room temperature. Finally, the proteins were detected with chemiluminescent substrate.

### Statistical analysis

All data were concluded from three independent experiments and shown as the mean ± SEM unless different indication. The data were analyzed by SPSS 19.0 software. One-way ANOVA was used to evaluate differences. *p* < 0.05 was considered significant differences.

## Electronic supplementary material

Supplementary Information
