# Publisher Correction: Effects of Thymoquinone on radiation enteritis in mice

**DOI:** 10.1038/s41598-021-83839-0

**Published:** 2021-03-24

**Authors:** Qinlian Hou, Linlin Liu, Yinping Dong, Jing Wu, Liqing Du, Hui Dong, Deguan Li

**Affiliations:** 1 Institute of Radiation Medicine, Chinese Academy of Medical Science and Peking Union Medical College, Tianjin Key Laboratory of Radiation Medicine and Molecular Nuclear Medicine, Tianjin, 300192 China; 2 Tianjin Hospital of Itcwm Nankai Hospital, Department of Pediatrics, Tianjin, 300100 China

Correction to: *Scientific Reports* 10.1038/s41598-018-33214-3, published online 11 October 2018

In the original version of this Article, Qinlian Hou and Linlin Liu were omitted as equally contributing authors.

This error has now been corrected in the HTML and PDF versions of the Article.

