# Supplementary material for: Effects of Thymoquinone on radiation enteritis in mice
Source: Sci Rep. 2018 Oct 11;8:15122. doi: 10.1038/s41598-018-33214-3 (PMC6181979; doi:10.1038/s41598-018-33214-3)
Supplement: Supplementary file 1 — Supplementary Information [file 41598_2018_33214_MOESM1_ESM.docx]

# Effects of Thymoquinone on radiation enteritis in mice

# Qinlian Hou^1#^, Linlin Liu^2#^, Yinping Dong^1^, Jing Wu^1^, Liqing Du^1^, Hui Dong^1^*, Deguan Li^1^*

SUPPLEMENTARY INFORMATION

**
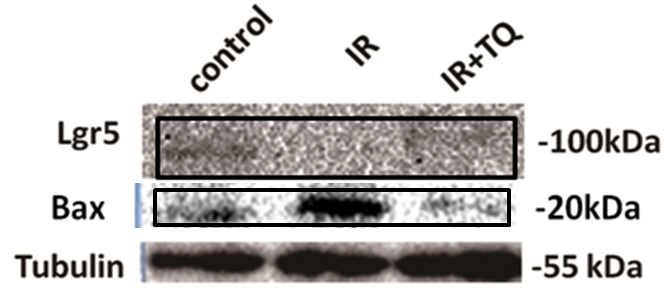
**

**SupplementaryFigure 1** The protein level of Lgr5 and Bax in small intestine was measured by Western blot.
